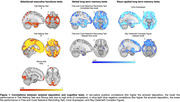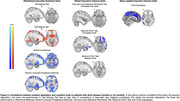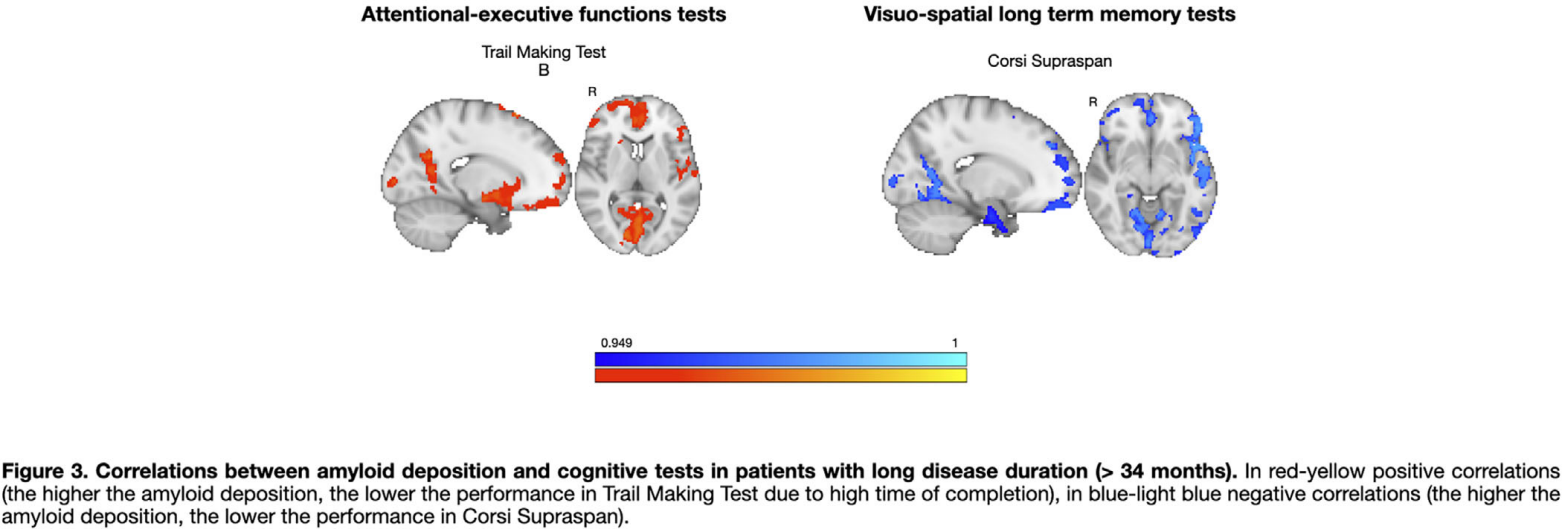# The relationship between amyloid‐b and cognition in young‐onset Mild Cognitive Impairment: the influence of disease duration

**DOI:** 10.1002/alz.090233

**Published:** 2025-01-03

**Authors:** Chiara Carbone, Erica Balboni, Daniela Beltrami, Manuela Tondelli, Chiara Gallingani, Federico Gasparini, Davide Salvatori, Simone Salemme, Giulia Vinceti, Alessandro Fraternali, Alessandro Marti, Annalisa Chiari, Giovanna Zamboni

**Affiliations:** ^1^ Università di Modena e Reggio Emilia, Modena Italy; ^2^ Fisica Medica, Azienda Ospedaliero Universitaria di Modena, Modena Italy; ^3^ Neuropsicologia Clinica, Disturbi cognitivi e Dislessia nell'adulto, Azienda Unità Sanitaria Locale di Reggio Emilia‐IRCCS, Reggio Emilia Italy; ^4^ Neurologia, Azienda Ospedaliero Universitaria di Modena, Modena Italy; ^5^ Scuola Internazionale di Studi Avanzati, Università di Camerino, Camerino Italy; ^6^ Medicina Nucleare, Azienda Unità Sanitaria Locale di Reggio Emilia‐IRCCS, Reggio Emilia Italy

## Abstract

**Background:**

The effect of amyloid‐b brain deposition on cognition is still debated, since it has been shown that its accumulation begins almost 15 years before cognitive symptoms' onset, then reaches a plateau while cognition continues to decline. We studied if there is a parallel between amyloid‐b deposition and cognitive performances in young‐onset Mild Cognitive Impairment (MCI) patients, and if it is associated to symptoms’ duration.

**Method:**

Subjects with a diagnosis of MCI and symptoms’ onset ≤ 65 years underwent neuropsychological assessment, Magnetic Resonance Imaging, and (^18^F)Flutemetamol‐PET (amy‐PET). FMRIB Software Library was used to assess voxel‐wise correlations between amyloid‐b deposition and cognitive performances, adjusted for age, sex, education, and degree of grey matter atrophy. Correlations were repeated grouping patients depending on whether the duration of their cognitive symptoms had been shorter or longer than the median of the entire group (34 months).

**Result:**

Among the 30 recruited individuals we found a statistically significant correlation between amyloid‐b burden and performance in attentional‐executive (i.e., Trail Making Test [TMT]‐A/B and Stroop Test) and memory (i.e., Free and Cued Selective Reminding Test [FCSRT]‐immediate and delayed free recall, Corsi Supraspan, and Rey‐Osterrieth Complex Figure‐delayed recall) tests.

Patients with short disease duration (n = 15) had even more significant associations relative to patients with longer duration. More precisely, they showed significant linear correlations in widespread brain regions between amyloid‐b burden and cognition in attentional‐executive (i.e., Attentional Matrices, Raven’s Coloured Progressive Matrices, TMT‐A/B) and memory (i.e., Babcock Short Tale‐immediate/delayed recall, FCSRT‐immediate free recall, and Corsi Supraspan) tests.

**Conclusion:**

In young‐onset MCI we found a linear relationship between amyloid‐b deposition and cognitive performance, which is more evident in patients with shorter disease duration. Our results contribute to identifying which cognitive tests are most sensitive to amyloid‐b deposition in the initial stages of dementing diseases.